# Prevalence of microalbuminuria and associated factors among HIV − infected ART naïve patients at Mulago hospital: a cross-sectional study in Uganda

**DOI:** 10.1186/s12882-020-02091-2

**Published:** 2020-10-20

**Authors:** Thomas Kiggundu, Robert Kalyesubula, Irene Andia-Biraro, Gyaviira Makanga, Pauline Byakika-Kibwika

**Affiliations:** 1 Department of Internal Medicine, School of Medicine, Makerere University College of Health Sciences, P.O. Box 7072, Kampala, Uganda; 2 Department of Internal Medicine, Uganda Martyrs Hospital, Lubaga, P.O. Box 14130, Kampala, Uganda; 3 Department of Physiology, School of Medicine, Makerere University College of Health Sciences, P.O. Box 7072, Kampala, Uganda; 4 Bank of Uganda, P.O. Box 7120, Kampala, Uganda; 5 Infectious Diseases Institute, Makerere University, P.O. Box 7072, Kampala, Uganda

**Keywords:** HIV, Microalbuminuria, Uganda, ART naïve

## Abstract

**Background:**

HIV infection affects multiple organs and the kidney is a common target making renal disease, one of the recognized complications. Microalbuminuria represents an early, important marker of kidney damage in several populations including HIV-infected antiretroviral therapy (ART) naïve patients. Early detection of microalbuminuria is critical to slowing down progression to chronic kidney disease (CKD) in HIV-infected patients, however, the burden of microalbuminuria in HIV-infected antiretroviral therapy (ART) naïve patients in Uganda is unclear.

**Methods:**

A cross-sectional study was conducted in the Mulago Immune suppression syndrome (ISS) clinic among adult HIV − infected ART naïve outpatients. Data on patient demographics, medical history was collected. Physical examination was performed to assess body mass index (BMI) and hypertension. A single spot morning urine sample from each participant was analysed for microalbuminuria using spectrophotometry and colorimetry. Microalbuminuria was defined by a urine albumin creatinine ratio (UACR) 30-299 mg/g and macroalbuminuria by a UACR > 300 mg/g. To assess the factors associated with microalbuminuria, chi-square, Fisher’s exact test, quantile regression and logistic regression were used.

**Results:**

A total of 185 adult participants were consecutively enrolled with median age and CD4+ counts of 33(IQR = 28–40) years and 428 (IQR = 145–689) cells/μL respectively. The prevalence of microalbuminuria was 18.9% (95% CI, 14–25%). None of the participants had macroalbuminuria. CD4+ count <350cells/μL was associated with increased risk of microalbuminuria (OR: 0.27, 95% CI: 0.12–0.59), P value = 0.001). Diabetes mellitus, hypertension, smoking, alcohol intake were not found to be significantly associated with microalbuminuria.

**Conclusion:**

Microalbuminuria was highly prevalent in adult HIV − infected ART naive patients especially those with low CD4+ count. There is need to study the effect of ART on microalbuminuria in adult HIV − infected patients.

## Background

The Joint United Nations Programme on HIV and AIDS (UNAIDS) estimates that at least 40 million people living with HIV globally and 2.1 million new infections occurring annually in 2014 [1]. Africa bears the world’s HIV burden of almost 30 million with majority (26.1 million) in Sub-Saharan Africa (SSA). In Uganda, HIV prevalence is estimated at 7.3% [1]. HIV infection affects multiple organs and the kidney is a common target making renal disease, one of the recognized complications [2–4].

Microalbuminuria represents an early, important marker of kidney damage and may predict the development of proteinuria, a feature of renal disease [5]. It is crucial, therefore, to identify microalbuminuria as soon as possible, particularly in this high risk population. The early detection is vital to slowing down progression to chronic kidney disease (CKD) among HIV-infected patients in a Ugandan setting where renal replacement therapy is not easily accessible.

The occurrence of microalbuminuria in HIV-infected antiretroviral therapy (ART) naïve patients has not been explored in Uganda. The purpose of our study was to determine the prevalence of microalbuminuria in this adult high risk population and assess any associated factors using a cross-sectional study.

## Methods

### Study design and setting

From March 2017 to April 2017, we conducted a cross-sectional study at Mulago ISS clinic, in Kampala, Uganda, which attends to a daily average of 5 new HIV − infected ART naïve patients. Mulago ISS clinic is an outpatient HIV-clinic at Mulago National Referral Hospital in Kampala, Uganda, which is run by Makerere University Joint AIDS Program (MJAP). Sample size estimation was guided by the using Kish Lesley (1965) formula, assuming a 5% precision, 1.96 level of confidence, previous prevalence of microalbuminuria of 14% in HIV [6], giving a size of 185.

### Ethics

Ethical approval was obtained from Makerere University School of Medicine Research and Ethics Committee as well as administrative permission from Mulago ISS clinic management to conduct the study. All patients provided a written informed consent to take part in the study.

### Participants

We consecutively enrolled adult out-patients who had a confirmed positive HIV serological test without prior ART. Research assistants stationed at Mulago ISS clinic from Monday to Friday provided information about the study and obtained informed consent prior to enrollment. We excluded patients with renal disease, hypertension, diabetes mellitus, menstruating women and pregnancy. An interviewer-administered structured questionnaire in a separate enrollment room within in the clinic was used to collect data on patient demographics, medical history. Physical examination was performed to assess body mass index (height, weight) and hypertension.

### Data collection and outcome measures

Blood samples were obtained for complete blood count, liver function tests (Aspartate aminotransferase [AST], Alanine aminotransferase [ALT], and albumin), serum urea, serum creatinine and CD4+ count.

Single spot morning urine specimens were obtained in the morning for urine albumin to creatinine measurement, transported in a cold chain to Mulago hospital laboratory and stored at − 80°c. Urine albumin and urine creatinine were measured using the SELECTRA ProXL® chemical analyzer (ELITech group solutions, France), utilizing spectrophotometry and quantitative method of colorimetric determination of albumin. Microalbuminuria was defined by a urine albumin creatinine ratio (UACR) 30-299 mg/g and macroalbuminuria by a UACR > 300 mg/g [5].

### Statistical analysis

The objective of this research was to determine the prevalence of microalbuminuria and associated factors among HIV − infected ART naïve patients at Mulago Hospital, Kampala, Uganda. For continuous variables i.e. age and CD4+ count the median and interquartile range were used to summarize the variables since these were not normally distributed. Categorical variables were summarized using frequencies and proportions. The chi-square and Fisher’s exact test were used to assess the association between the categorical variables and outcome. Conversely, quantile regression was used to assess the association between the categorical variables and outcome i.e. microalbuminuria. Variables that were found to have an association with the outcome were then considered for the subsequent logistic regression analysis except for age and sex. Univariate logistic regression was used to estimate the effect of each explanatory variable on microalbuminuria. Crudes odds ratios were then obtained and reported. Multivariate logistic regression models were fitted to estimate the effect of the covariates on the outcome (microalbuminuria) adjusting for each other and for potential confounders, i.e. age, sex, CD4+ count and BMI categorized. Each of the explanatory variables was added one at a time and those that were found to be independently associated with the outcome were then left in the model. For the variables that were not independently associated with the outcome were then added back in the model to assess whether there was an association between these variables and the outcome in the presence of other variables. A final model including age, sex and CD4+ count was then fitted. The Hosmer-Lemeshow test was then used to test for goodness of fit of the final model and the P-value (0.437) showed no evidence to reject the null hypothesis that the fitted model was correctly specified. For this particular test 20 groups were specified, and explored observed versus expected values. After fitting the final model, adjusted ORs and 95% CIs were obtained and reported. All tests were done at 5% level of significance and analysis was done using Stata version 15.

## Results

### Characteristics of participants

A total of 185 participants were enrolled in the study. For these participants, the median age and CD4+ counts were 33(IQR = 28–40) years and 428 (IQR = 145–689) cells/μL respectively. 63.8% of them were female and 91.9% were employed in the informal sector. 6.5, 51.3, and 61.1% of the participants were smokers, had a history of alcohol consumption, and had normal weight respectively.

The prevalence of microalbuminuria among these participants was 18.9% (95% CI: 14–25%). None of the participants had macroalbuminuria. Most (60%) of participants with microalbuminuria were also single and had primary education attained as their highest level of education. Most of these participants, 68.8%, had a CD4 + count of < 350 cells/μL. (Table 1).

**Table 1:** Baseline demographic and clinical characteristics of study participants (*N* = 185)

Variable	Normal UACR	Microalbuminuria	Total	***P***-value
**Age in years, median(IQR)**	33 (28–40)	34 (26–40)	33 (28–40)	0.658
**CD4+ count (cells/μL), median(IQR)**	460 (215–731)	215 (84–539)	428 (145–689)	0.007
**Sex**				
Male	59 (39.3)	8 (22.9)	67 (36.2)	
Female	91 (60.7)	27 (77.1)	118 (63.8)	0.080*
**Occupation**				
Informal	137 (91.3)	33 (94.3)	170 (91.9)	
Formal	13 (8.7)	2 (5.7)	15 (8.1)	0.740*
**Highest level of education**				
None	14 (9.3)	0 (0.0)	14 (7.6)	
Primary	70 (46.7)	21 (60.0)	91 (49.2)	
Secondary	49 (32.7)	13 (37.1)	62 (33.5)	
Tertiary	17 (11.3)	1 (2.9)	18 (9.7)	0.079*
**Marital Status**				
Married	58 (38.7)	10 (28.8)	68 (36.8)	
Single	77 (51.3)	21 (60.0)	98 (53.0)	
Widowed	6 (4.0)	0 (0.0)	6 (3.2)	
Others	9 (6.0)	4 (11.4)	13 (7.0)	0.327*
**History of Alcohol consumption**				
Yes	76 (50.7)	19 (54.3)	95 (51.3)	
No	74 (49.3)	16 (45.7)	90 (48.7)	0.700
**History of smoking**				
Yes	9 (6.0)	3 (8.6)	12 (6.5)	
No	141 (94.0)	32 (91.4)	173 (93.5)	0.701*
**BMI in Kg/m**^**2**^				
Underweight	12 (8.0)	9 (25.7)	21 (11.4)	
Normal	93 (62.0)	20 (57.1)	113 (61.1)	
Overweight	26 (17.3)	5 (14.3)	31 (16.8)	
Obese	19 (12.7)	1 (2.9)	20 (10.8)	0.020*

*P*-value*: fishers exact p-value

Majority of participants were Christians, not on any on any concomitant medications, did not have hypertension or diabetes mellitus (79.5, 78.4, 9.2, and 18.9% respectively). There were no statistically significant differences in the mean urea and creatinine among participants with normal UACR and microalbuminuria {2.5 (0.5) mmol/L and 59.1 (16.0) μmol/L versus 2.8 (1.1) and 62.3 (28.4)} (p value 0.3 and 0.08) respectively.

### Factors associated with microalbuminuria

From the multivariate model: There was solid evidence (*P* = 0.001) of an association between CD4+ count and microalbuminuria after adjusting for sex and age in the model. Participants with CD4+ count greater than or equal to 350 cells/μL had lower odds of having this condition as compared to those with CD4+ count less than 350 cells/μL. (OR:0.27, 95% CI: 0.12–0.59). Also, there was some evidence of an association between sex and microalbuminuria. Females had higher odds of developing this condition as opposed to male participants. (OR: 2.61, 95% CI: 1.03–6.61). There was no association (*P* = 0.292) between age and microalbuminuria after adjusting for sex and CD4+ count in the model Table 2.

**Table 2:** Factors associated with microalbuminuria

Variable	Univariate model			Multivariate model		
	Crude OR	95% CI	***P***-value	Adjusted OR	95% CI	***P***-value
**Age in years**	1.01	0.97–1.06	0.503	1.02	0.98–1.07	0.292
**Gender**						
Male	ref	ref		Ref	ref	
Female	2.18	0.93–5.14	0.072	2.61	1.03–6.61	0.043
**CD4+ count (cells/μL)**						
< 350	ref	ref		ref	ref	
≥ 350	0.27	0.12–0.58	0.001	0.27	0.12–0.59	0.001
**Grouped BMI, kg/m**^**2**^						
Normal	ref	ref				
Underweight (< 18.5)	3.49	1.30–9.39				
Overweight (25–30)	0.89	0.31–2.61				
Obese (30+)	0.24	0.03–1.94	0.027			

*OR* Odds Ratio, *95% CI* 95% Confidence Interval, *BMI* Body Mass Index. Adjusted model: Adjusted for age, sex, and CD4+ count (cells/μL)

## Discussion

The prevalence of microalbuminuria in HIV − infected ART naïve patients in this study was 18.9% and was associated with low CD4+ count. Microalbuminuria has been shown to be one of the early markers for renal injury and an important predictor for both morbidity and mortality in HIV − infected patients [5, 7]. This study has provided the prevalence of microalbuminuria and its associated factors which had not been well established among ART naïve adult patients in Uganda.

This study looked at HIV − infected ART naïve patients attending the Mulago ISS clinic and the prevalence of microalbuminuria reported was different from most of the studies conducted in recent times [5, 6, 8–14]. It was challenging to compare this study with those conducted earlier due to several factors which include differing study participants and sample sizes, in addition to varying assay techniques and methodologies.

The most recent and similar study done in Tanzania [14] found prevalence at 20.4% which is close to that demonstrated by our study (18.9%) though it assessed HIV − infected children. However, a study from South Africa [8] found the prevalence of microalbuminuria at 24.0%. They included in-patients, who are known to be at higher risk of microalbuminuria due to advanced HIV [15] compared to ambulatory patients and this could explain the higher prevalence. Another study [13] demonstrated that advanced HIV disease was significantly associated with microalbuminuria. They found a higher prevalence of microalbuminuria at 28.8% but included participants on ART. In the same study, they demonstrated that recent ART was associated with microalbuminuria. This too was demonstrated in USA [6] that patients who were recently ritonavir were more likely to have microalbuminuria compared to those who were not on ritonavir (19.8 vs.7.4%).

The only study done in Africa that looked at participants with similar characteristics (adult HIV ART naïve patients attending an outpatient clinic) was in Tanzania [10] demonstrated the higher prevalence of microalbuminuria at 70.2%. In this study, they used dipsticks and the patients had relatively lower mean CD4+ count compared to our study (200 versus 466 cells/μL).

Notably, there were two studies in adults that demonstrated lower prevalence 8.9% [12] in Democratic Republic of Congo(DRC) and 14% [6] in USA having included both ART experienced and ART naïve patients. Other studies that have found lower prevalence (less than 19% in our study) were conducted among HIV − infected Nigerian children reported prevalence of microalbuminuria of 11.1 and 12% [9, 11]. Overall, the differences in screening methodologies and the clinical characteristics of study participants probably explain the varied results among studies in relation to our study.

### Factors associated with microalbuminuria

We found that low CD4+ count (<350cells/μL) was independently associated with microalbuminuria. Several studies have shown microalbuminuria being associated with reducing CD4 + counts. Our study looked at CD4+ count as continuous variable with microalbuminuria being associated with lower mean CD4+ count compared to normal UACR (320 versus 500cells/μL). A prospective cohort study [6] demonstrated that those with microalbuminuria were more likely to have a CD4 count < 200 cells/μL. In our study, when CD4 + count was analysed as a categorical variable, those with microalbuminuria were more likely to have CD4 + count < 350 cells/μL even after adjusting for age and sex. The scientific explanation for the relationship between microalbuminuria and low CD4 + counts has not been fully explained. It is, however, likely that our study participants who had low CD4 + counts did have more advanced disease and likely to have had early or even overt HIVAN.

The relationship between microalbuminuria and underweight has not been established among adult HIV − infected patients. In our study, microalbuminuria was associated with being underweight, a finding that was evident in other populations [13, 16, 17]. However, our study may not have been powered enough to ascertain these relationships.

### Limitations

Although our study did not find significant association between other factors such as hypertension, diabetes mellitus age, sex, alcohol use and smoking with microalbuminuria, we did not assess the association between microalbuminuria and viraemia due to financial constraints yet it has been cited as a predictor of microalbuminuria [5]. Hypertension was defined by two blood pressure readings of a systolic ≥140 mmHg and diastolic ≥90 mmHg taken 30 min apart. This was not the best method but the most feasible for an out-patient clinic.

## Conclusion

The prevalence of microalbuminuria in HIV − infected ART naïve patients was 18.9% and the associated low CD4+ count. This study has enlightened the magnitude of the earliest form of renal dysfunction in HIV − infected ART naïve patients and therefore screening for microalbuminuria should be advocated for before ART initiation.

## Recommendations

HIV − infected patients with CD4+ count < 350 cells/μL may need to be screened for microalbuminuria before ART initiation.

There is need for further assessment of the relationship between underweight and microalbuminuria in a larger study.

There is need for further assessment of the progression of microalbuminuria in HIV patients after initiation of ART.

## Supplementary information

**Additional file 1.** Questionnaire.

**Additional file 2.** Informed consent form.

## Data Availability

All the data supporting the findings is submitted with the manuscript.

## Abbreviations

ALT: Alanine aminotransferase
AST: Aspartate aminotransferase
ART: Antiretroviral therapy
BMI: Body mass index
CD: Cluster of differentiation
DRC: Democratic Republic of Congo
HIV: Human immunodeficiency virus
ISS: Immune suppression syndrome
MJAP: Makerere University Joint AIDS Program
UACR: Urine albumin creatinine ratio
UNAIDS: The Joint United Nations Programme on HIV and AIDS
USA: United States of America
