# Supplementary material for: Prevalence of microalbuminuria and associated factors among HIV − infected ART naïve patients at Mulago hospital: a cross-sectional study in Uganda
Source: BMC Nephrol. 2020 Oct 20;21:440. doi: 10.1186/s12882-020-02091-2 (PMC7574295; doi:10.1186/s12882-020-02091-2)
Supplement: Supplementary file 1 — Additional file 1. Questionnaire. [file 12882_2020_2091_MOESM1_ESM.docx]

## Additional file 1: QUESTIONNAIRE

Thank you for accepting to be part of this study.

It is to find out the magnitude of patients who have HIV and are at risk of kidney disease by using urine protein (micro-albuminuria). All the information given will be kept confidential and only used for this research purposes. Your participation will not affect the care you receive from here.

**SECTION A: IDENTIFICATION**

1. Study number
2. Address
3. Telephone contact

**SECTION B: SOCIAL DEMOGRAPHIC AND FAMILY CHARACTERISTICS**

1. Age ____________
2. Sex: 1 Male 2. Female
3. LNMP(for females only)_____________________
4. Occupation
5. Level of education: 1. None 2. Primary 3. Secondary 4. Tertiary
6. Marital status: 1 Married 2. Single 3. Widowed 4. Others_____________
7. Religion 1. Catholic 2. Moslem 3. Anglican 4. Saved 5. SDA 6. Others_______________________
8. Tribe
9. History of any alcohol use 1. No 2. Yes
10. If yes, do you still take? 1. No 2. Yes
11. If yes, how much per sitting?
12. Duration?
13. History of any smoking 1. Yes 2. No
14. If yes, do you still smoke?
15. How often per day?
16. Duration?
17. History of Diabetes 1. Yes 2.No
18. If yes, duration?
19. What medications
20. History of Hypertension 1.Yes 2.No
21. If yes, duration?
22. What medications__________________________
23. History of any other medications 1.Yes 2.No
24. If yes, what medications
25. Weight (kg)
26. Height (m)
27. BMI
28. Blood pressure
29. CBC: WBC
30. CBC: Hb
31. CBC: MCV
32. CBC:PLT

RFT

1. RFT: Creatinine
2. RFT: Urea

LFT

1. LFT: Albumin
2. LFT:AST
3. LFT: ALT
4. LFT: GGT
5. LFT: Bilirubin(T)
6. LFT: Bilirubin(D)
7. CD4^+^ count
8. Viral load
9. Urine albumin
10. Urine Creatinine
11. Urine albumin creatinine ratio
