# Supplementary material for: Prevalence of microalbuminuria and associated factors among HIV − infected ART naïve patients at Mulago hospital: a cross-sectional study in Uganda
Source: BMC Nephrol. 2020 Oct 20;21:440. doi: 10.1186/s12882-020-02091-2 (PMC7574295; doi:10.1186/s12882-020-02091-2)
Supplement: Supplementary file 2 — Additional file 2. Informed consent form. [file 12882_2020_2091_MOESM2_ESM.docx]

## Additional file 2: INFORMED CONSENT FORM

### English version of the informed consent form

**Title of the study**: Prevalence of Microalbuminuria and associated factors among ART naïve HIV patients at Mulago hospital, Kampala, Uganda.

**Name of Principal Investigator**: I am Dr.Thomas Kiggundu, a Senior House officer at the Department of Internal medicine Makerere University College of Health sciences, Mulago Hospital. Email: dr.kiggundu@gmail.com, phone number +256-752-457453

**Introduction**

HIV infection affects multiple organs and the kidney is a common target and as such, Microalbuminuria (passing protein in urine) has been showed to predict the development of kidney disease among HIV-infected persons. There is little available evidence about the magnitude of microalbuminuria in HIV−infected ART naïve patients in our local setting; this study will measure the prevalence and determine factors associated with microalbuminuria. This data will provide information for HIV care programs to guide management of HIV infected patients

You are invited to participate in a study whose details are stated here in.

**Purpose**

I would like to assess the occurrence of Microalbuminuria (passing protein in urine) and its factors associated factors among HIV−infected ART naïve patients at Mulago hospital, Kampala, Uganda

**Participant selection/Purpose**

You have been selected for the study because you have HIV infection, above 18 years of age and you have not yet started taking the HIV drugs. You also qualified because you are not pregnant for females.

Procedures

You will be requested to provide a sample of your urine to assess protein in it. Blood samples, however, will be taken off for function of the Liver, Kidney and immune status as routine for all new patients coming into the clinic. The results of the urine test will be given to you on the next review, and if you have any abnormality you will be guided accordingly.

**Voluntary Participation**

This participation is voluntary. You can choose to participate or not. You will still receive all the services that you usually do whether you choose to participate or not. You can also choose to stop your participation any time during the study.

**Discomforts**

You will be asked to provide some urine and blood. You may feel slight discomfort in terms of pain while being pricked to draw blood and delay while providing the urine for the study.

**Benefits**

The test we are going to perform will be able to show us whether you are at a high risk of kidney disease or not. This test will be carried out at no cost to you.

**Incentives**

The tests however will be paid for by the study and Mulago ISS clinic.

**Confidentiality**

The information gathered will only be available to the research team and your attending health workers.

**Sharing the Results**

The result of the test will be given to you on the next clinic review. At the end of the study, we will present what the study found regarding kidney disease in HIV to the clinic and hospital workers. You will be invited by telephone for this presentation if you wish to attend. You have a right to refuse or withdraw your participation at any time.

**Who to Contact**

For further enquiries contact: Dr Thomas Kiggundu, Senior House officer. Department of Medicine Makerere University College of Health sciences Mulago Hospital Email: [dr.kiggundu@gmail.com](mailto:dr.kiggundu@gmail.com), phone number +256-752-457453

**Questions about participants’ rights**

In case you have questions regarding your rights as a research participant, you may contact the Chairman of the School of Medicine Research Ethics Committee, Assoc. Prof. Ponsiano Ocama Telephone +256-772-421190.

**PART II: Statement of Consent /Assent**

………………………………………………………………has described to me what is going to be done, the risks, the benefits involved and my rights regarding this study. I understand that my decision to participate in this study will not alter my usual medical care. In the use of this information, my identity will be concealed. I am aware that I may withdraw at any time. I understand that by signing this form, I do not waive any of my legal rights but merely indicate that I have been informed about the research study in which I am voluntarily agreeing to participate. A copy of this form will be provided to me

Name ………………………………………………

Signature of participant …………………………………… Date …………………

Name ……………………..........................

Signature of interviewer …………………………………. Date ……………………
